# 140 Development of public facing physical activity messages to accompany the updated National Physical Activity and Sedentary Behaviour Guidelines for Ireland

**DOI:** 10.1093/eurpub/ckae114.057

**Published:** 2024-09-26

**Authors:** Chloë Williamson, Dylan Power, Elaine Murtagh

**Affiliations:** Physical Activity for Health Research Centre, The University Of Edinburgh, Edinburgh, United Kingdom; Department of Physical Education & Sport Sciences, University of Limerick, Limerick, Ireland; Department of Physical Education & Sport Sciences, University of Limerick, Limerick, Ireland

## Abstract

**Purpose:**

One assumption that has been made previously is that physical activity (PA) guidelines themselves double up as effective PA messages, yet PA guideline knowledge remains low. This study aimed to develop public-facing messages to accompany the Updated National Physical Activity and Sedentary Behaviour Guidelines for Ireland (forthcoming – March 2024).

**Methods:**

For each guideline population subgroup (n = 6), available evidence relating to concepts within the Physical Activity Messaging Framework was used to develop two messages sets with distinct aims. Using the Behaviour Change Wheel, set 1 utilised education, aiming to increase knowledge. Set 2 utilised persuasion, aiming to improve perceptions, attitudes, and motivation. Messages were shared with the public in an online survey comprising Likert scale questions to assess clarity and relevance (set 1) and motivation and appeal (set 2), and open-ended questions to gain feedback on messages. Feedback was discussed at a Stakeholder Consensus Meeting which informed consensus on final messages for each subgroup.

**Results:**

The survey received 215 public responses (70% female). Overall, there was strong agreement that message sets 1 and 2 were clear, relevant, motivational, and appealing to the reader. For each subgroup, consensus was reached on a bank of messages for sets 1 and 2. For message set 1, a preference for presenting guidelines as hours and minutes (e.g., 2 hours and 30 minutes) as opposed to minutes (e.g., 150 minutes) was identified. For message set 2, top ranked messages focused on shorter term benefits of PA such as mental and social health outcomes. “Every move is a good move” and “Every move counts” were proposed as overall taglines to link messages.

**Conclusions:**

This was the first study to apply the PAMF prescriptively to develop public facing PA messages concurrent with the development of updated national PA guidelines. This process allowed for a bank of evidence-based and targeted PA messages for dissemination in Ireland for 6 population subgroups. In line with the Global Action Plan for Physical Activity 2018-2030, these messages have the potential to change PA knowledge, perceptions, and attitudes, and contribute to promoting population level health-enhancing PA.

**Funding Source:**

Health Service Executive (HSE).

